# Proposed protocol for post COVID-19 cognitive rehabilitation for attention and memory

**DOI:** 10.1590/1980-5764-DN-2023-0109

**Published:** 2024-05-27

**Authors:** Letícia Silva Dutra, Nadia Shigaeff

**Affiliations:** 1 Universidade Federal de Juiz de Fora, Grupo Interdisciplinar de Pesquisa em Neuropsicologia e Gerontologia, Juiz de Fora MG, Brazil.

**Keywords:** Cognitive Training, Cognitive Dysfunction, COVID-19, Treino Cognitivo, Disfunção Cognitiva, COVID-19

## Abstract

Since the beginning of the COVID-19 pandemic, many people suffered from Long Covid Syndrome, in which affected individuals do not recover immediately after the end of the infectious and inflammatory process caused by the virus. The most common neuropsychological symptoms of this syndrome are: memory decline, lack of attention, anxiety and depression.

**Objective:**

The purpose of this study was to develop a proposed cognitive rehabilitation protocol for post-COVID individuals with cognitive symptoms.

**Methods:**

A rehabilitation proposed protocol focusing on attention and memory was developed, based on the tests used in the neuropsychological evaluation of affected patients. Researchers held weekly sessions for six months, each lasting 60 minutes. Homework activities were also assigned and corrected in the following session. The attention and memory sessions were conducted with activities based on the applied tests.

**Results:**

Despite the methodological separation of attention and memory, the activities indirectly affect other cognitive functions and abilities, such as executive function, language, reasoning, execution strategies, and cognitive flexibility. A computer, a sheet of paper, and a pen were used to present the slides for the activities. Attention training included all types of attention: sustained, alternating, selective and divided. Memory training sessions included activities that stimulated both short-term and long-term memory. With each session, the difficulty of the activities was gradually increased.

**Conclusion:**

Cognitive rehabilitation already has more consolidated evidence about its effectiveness for the treatment of other pathologies, so it can be thought that it will also be a promising strategy for COVID-19 too.

## RESUMO

Desde o início da pandemia de COVID-19, muitas pessoas sofreram com a síndrome da COVID longa, em que os indivíduos afetados não se recuperam após o término do processo infeccioso e inflamatório causado pelo vírus. Os sintomas neuropsicológicos mais comuns dessa síndrome são: declínio da memória, falta de atenção, ansiedade e depressão.

**Objetivo:**

Desenvolver uma proposta de protocolo de reabilitação cognitiva para indivíduos pós-COVID com sintomas cognitivos.

**Métodos:**

Foi desenvolvida uma proposta de protocolo de reabilitação com foco na atenção e na memória, baseada nos testes utilizados na avaliação neuropsicológica dos pacientes afetados. Os pesquisadores realizaram sessões semanais durante seis meses, cada uma com duração de 60 minutos. As atividades de lição de casa também foram atribuídas e corrigidas na sessão seguinte. As sessões de atenção e memória foram realizadas com atividades baseadas nos testes aplicados.

**Resultados:**

Apesar da separação metodológica entre atenção e memória, as atividades afetam indiretamente outras funções e habilidades cognitivas, como função executiva, linguagem, raciocínio, estratégias de execução e flexibilidade cognitiva. Foram utilizados computador, folha de papel e caneta para a apresentação dos *slides* das atividades. O treinamento da atenção incluiu todos os tipos de atenção: sustentada, alternada, seletiva e dividida. As sessões de treinamento de memória incluíram atividades que estimularam memória de curto e longo prazo. A cada sessão, a dificuldade das atividades foi aumentando gradativamente.

**Conclusão:**

A reabilitação cognitiva já possui evidências mais consolidadas sobre a sua eficácia para o tratamento de outras patologias, pelo que se pode pensar que na COVID-19 será também uma estratégia promissora.

## INTRODUCTION

The COVID-19 pandemic, as per the World Health Organization, has resulted in over six million deaths and infected more than 700 million individuals worldwide^1^. This has also given rise to Long Covid Syndrome, characterized by lingering symptoms following the acute phase, i.e., even after the infection has been resolved^2^. Notable neuropsychological manifestations encompass memory issues, cognitive difficulties, brain fog, and anxiety, with memory and attention problems recurring most frequently^3^.

Given the cognitive challenges post-infection, it is crucial to explore therapeutic approaches to enhance quality of life. Cognitive rehabilitation offers a promising strategy for individuals with cognitive impairments^4^. This approach can be facilitated through various methods, including paper-pencil tools, computer programs, and virtual reality (VR)^5^.

While the “Evidence-Based Cognitive Rehabilitation” review^6^ provides valuable insights, it predates the COVID-19 pandemic, necessitating dedicated research for post-COVID cognitive issues. Detailed rehabilitation protocols are scarce, and this study aims to fill that gap, aiding researchers, professionals, and students in cognitive rehabilitation interventions focusing on attention and memory.

## METHODS

This proposed protocol is a part of the research project “Clinical trial on the effect of cognitive rehabilitation in individuals with COVID-19”, approved by the Research Ethics Committee under registration Certificate of Presentation for Ethical Appreciation (CAAE) 47489021.2.0000.5147 and registered with the International Clinical Trials Registry Platform — ICTRP/WHO and Brazilian Registry of Clinical Trials (ReBEC) under protocol RBR-967k5py.

Participants underwent neuropsychological assessments that highlighted memory and attention deficits, which were targeted in the rehabilitation program. Randomization divided participants into control and experimental groups. The experimental group underwent a six-month cognitive rehabilitation program, meeting weekly for 60-minute sessions, with mild and moderate to severe cognitive impairment groups.

The protocol presented here is an important contribution, as it departs from VR-based rehabilitation approaches, having a low cost and being more accessible. It was developed specifically for this study due to the dearth of detailed rehabilitation protocols. The assessment tools used were the Hopkins Verbal Learning Test/Revised^7^ for episodic long-term memory, the Trail Making Test^8^ and Stroop Test (Victoria version)^9,10^ for attention, and various other tests to assess executive function, language, and information processing speed^11^. This rehabilitation protocol was designed based on assessments revealing impaired performance in memory and attention tests.

## RESULTS

The activities used throughout the rehabilitation protocol were based on the tests applied in the evaluation of patients, already mentioned in the methods item. Sessions related to attention and memory were performed, with a 2x1 ratio, respectively. This proportion was decided on the basis of the test results of the study’s patients, who showed a greater degree of cognitive impairment in the attention domain. It is important to point out that, despite this methodological separation of attention and memory, the proposed activities indirectly train other cognitive functions and abilities, such as executive function, language, reasoning, execution strategies, cognitive flexibility, among others. Furthermore, even attention focused sessions stimulate memory indirectly, and vice versa, as it is not possible to stimulate only an isolated cognitive function.

The sessions were held at the Federal University of Juiz de Fora in groups of no more than four participants, with efforts made to group individuals with similar levels of difficulty, and, when possible, similar age and educational backgrounds. A computer was used to present figures and slides related to the activities and sheets of paper and pen for each participant. Each session lasted approximately 60 minutes, divided among activities during the session and correction of homework, besides some moments of conversation and relaxation among participants.

In the first sessions, a psychoeducation strategy was carried out with the participants, based on conversations about how rehabilitation is possible due to neuroplasticity and also about our brain being trainable, so that we can learn and improve new skills. This explanation was considered necessary because the protocol activities are repetitive, and despite the increasing level of difficulty throughout the process, the commands are often the same. Therefore, it was also important to psychoeducate about the importance of this repetition during the rehabilitation process. It is known that it is important to explain to the participants what the aim of each activity is, so that there is greater adherence to treatment.

In the attention sessions, all types of attention were trained in the same session: sustained, alternating, selective and divided, respectively. In the memory sessions, activities were performed that stimulated both working (operational) memory and long-term memory. At each session, the difficulty of the activities was gradually increased. When starting the protocol, the participants had great ease with the proposed activities, which started in a simpler way. Some sessions were then anticipated, so that the degree of difficulty increased and the activities were more coherent, in order to stimulate the participants according to their performance.

### Session activities

**Figure 1:**
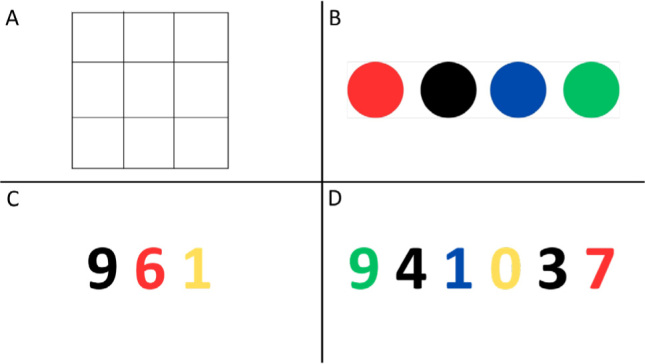
Examples of activities for attention.

To stimulate sustained attention during the session, the participants were presented with images containing several geometric figures, some inside others, and each participant was asked to write down the number of figures found on a piece of paper. After completing the activity, the review was carried out, and participants were corrected in case of errors by pointing out the missing geometric shapes. Figure 1A is an example of a sustained attention activity: How many squares are there in the figure? The correct answer would be 14 squares.

To facilitate alternating attention, we devised an oral activity inspired by the Trail Making Test. The facilitators also joined in, necessitating an odd number of participants for the training activity, ensuring that participants alternated between providing answers involving numbers and words. The objective of the activity was for each participant to verbalize a number (in ascending order), followed by the next participant providing a word (in alphabetical order) related to the chosen theme, and so forth. The themes varied according to the level of difficulty. In the initial sessions “proper names” were used, which was a consensus among the participants to be easier to remember. In the more advanced sessions, themes such as “clothing”, “cities”, “food”, among others, were used.

Example within the theme “proper names” with participants named X, Y and Z: Participant X-One; Participant Y-Alex; Participant Z-Two; Participant X-Bruna; Participant Y-Three; Participant Z-Carol; Participant X-Four; Participant Y-Daniel; Participant Z- Five, and so on. In this way, the participants alternated among numbers in ascending order and proper names, with the first letter of the name following the alphabetical order.

When the participants began to find this activity easy, changes were implemented to make the activity more difficult. Another example was the change from one theme to two. Thus, in addition to switching among numbers and words, within the category of word, they should alternate between the themes “object” and “country”.

Examples using the themes “object” and “country” with the participants named X, Y and Z: Participant X-One; Participant Y-Closet; Participant Z-Two; Participant X-Brazil; Participant Y-Three; Participant Z-Chair; Participant X-Four; Participant Y-Denmark; Participant Z-Five, and so on.

To stimulate selective attention, tasks were used that changed, on average, every four sessions, based on the Stroop Color and Word Test. In the initial sessions, slides with colored circles and the following instruction were presented: “Say out loud the color that is inside each circle, but whenever a blue circle appears, you must say red.” Then approximately 15 slides with circles of different colors were presented. Figure 1B is an example slide showing colored circles. The correct answer would be: red; black. red; green.

At each session, the color of the instruction was changed, and more circles appeared each time, to increase the degree of difficulty. In later sessions, the activity changed a little, and the instruction became: “Name the colors in which the numbers are written in as quickly as possible, but whenever a black number appears, it is the number itself that must be read.” Figure 1C is an example of a slide showing colored numbers in the beginning sessions. The correct answer would be: nine; red, yellow. And in Figure 1D the correct answer would be: green; four; blue; yellow; three; red.

In the memory sessions, activities were performed that stimulated short-term memory (immediate and working memory) and episodic long-term memory. At the beginning of the session, the participants were asked to visualize an image for approximately one minute. After that time, they were asked to reproduce it with pen on paper, but without having access to the image at that time, thus stimulating the immediate short-term memory for visual material. It was explained that the artistic abilities of each one would not be evaluated, but the details and elements that they could remember would.

**Figure 2:**
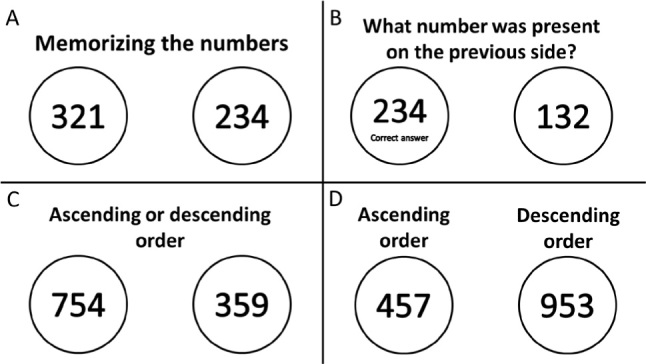
Examples of activities for memory.

Then a new activity was presented as a distractor, with the following instruction: “A sequence of numbers will be presented per slide. On the next slide, you need to discriminate which number was present on the previous screen.” To increase the degree of difficulty, larger numbers were presented, with more digits to remember. Figures 2A and 2B are examples of two slides showing numbers to remember which were present on the previous slide.

This activity varied throughout the sessions. Another example had the following command: “A numerical sequence will be presented and you must place the sequence in ascending/descending order”; in this way, the activity would require mental manipulation, training the working memory. The degree of difficulty was increased by placing more numbers on each slide. Figures 2C and 2D are examples of two slides showing numbers and commands (ascending or descending) for the participants to remember, manipulate and provide a response later on by quickly viewing this slide.

**Figure 3:**
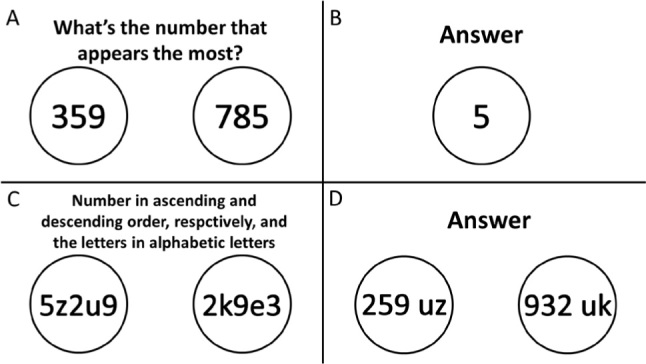
Examples of activities for memory.

A third variation had the following command: “A sequence of numbers will be presented per slide. You need to determine which number appears the most per slide.” The degree of difficulty was increased by placing more numbers on each slide. Figures 3A and 3B are examples of two numbers; participants should answer “5” as the number that appears the most when quickly viewing this slide at a later time.

Finally, the last and most complex variation was mixing numbers and letters. The participants had to place the numbers in ascending and descending order, respectively, and the letters in alphabetical order, at which point the image would no longer appear on the screen. Figures 3C and 3D are examples of numbers mixed with letters, and participants should answer “259 UZ 932 EK”.

After completing this activity, the previous one was resumed, referring to image reproduction. The participants were asked to redraw the image they previously drew, to stimulate episodic long-term memory for visual material. Approximately 20 minutes after the first stimulation, they were prompted to redraw the image. Once completed, a “correction” and a comparison were made between the images drawn in the two different moments, so that the participants could perceive the differences and the details that they forgot to include in the drawing itself.

**Figure 4:**
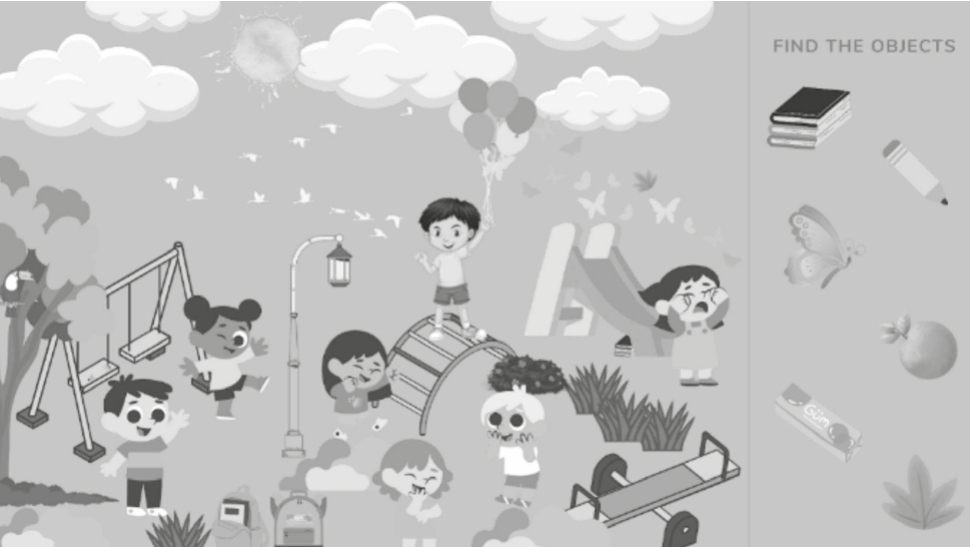
Example of activity to find objects.

Therefore, an episodic long-term memory task was presented, with verbal stimulation, in which participants should pay attention to the words that were said verbally, with an average of eight to 15 words. Then, a task was presented as a distracting stimulus, which stimulated attention, and which could be a seven errors game or a task of finding objects in an image (Figure 4).

After the distracting task, questions were asked with the aim of stimulating delayed recall. Some examples are questions related to words mentioned before the distracting activity, such as “Which words were types of food?”, and “What words started with the letter F?”. After this activity, a recognition task was performed using verbal stimuli, in which the previously spoken words were mixed with new distracting words, that is, words that had not been presented before, totaling 15 to 20. These were said orally and the participants had to write on a piece of paper what they thought would be the original words.

Finally, the session ended and the homework tasks were handed in. Throughout the session there was an opportunity to ask questions and make corrections, so that participants were aware of their own performance. However, this was done with the constant reminder that those were training sessions, so the purpose of activities was for them to learn, not be tested.

### Activities for home

**Figure 5:**
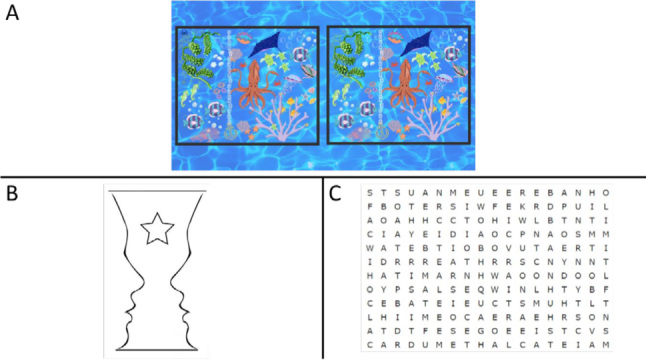
Examples of activities for homework.

The activities to be done at home were delivered in printed form, weekly, at the end of each session. The task was composed of four different types of activities, training all types of attention and memory. To train sustained attention, the “seven errors game” was used (Figure 5A). To train alternating attention, Gestalt therapy images (figure and background), an approach derived from psychology, were used (Figure 5B). To train selective attention, word searches were performed, with different levels of difficulty, some with word clues and others without these clues as to which words to find (Figure 5C). In the memory sessions, participants were asked to play the memory game at home, whether on the computer, cell phone or physical game.

## DISCUSSION

### Cognitive rehabilitation

Cognitive rehabilitation is aimed to improve the individual’s everyday functioning and should include active attempts to promote generalization or directly apply compensatory strategies to functional contexts^6^. At the time of writing the present study, there was one published scientific study (a letter to the editor) on cognitive rehabilitation for Long Covid, so it is not possible to have sufficient evidence to prove its effectiveness^12^.

The study implemented an eight-week outpatient neurorehabilitation program for post-COVID-19 syndrome patients, incorporating respiratory, physical therapy, and neuropsychological rehabilitation using the Guttmann NeuroPersonalTrainer^®^ platform. The study involved 50 patients without prior neurological conditions who completed the program between June 2020 and January 2021. Significant improvements were observed in memory tasks for all patients, and hospitalized patients also showed enhanced executive control. Anxiety and depression symptoms decreased post-intervention. Despite the absence of a control group, the study suggests the efficacy of neuropsychological rehabilitation in treating cognitive and mood disorders in post-COVID-19 syndrome patients, underscoring the need for further research with longer follow-up periods^12^.

Still according to the aforementioned review^6^ and the authors of the present proposed protocol, it is possible to perceive a trend in the use of cognitive rehabilitation based on VR, which is consistent with the increasingly rapid advancement of technology. However, the equipment used for this type of training is generally less accessible, which can make it difficult or even impossible to treat some individuals. Therefore, it is important that there are low-cost strategies that can be implemented with less investment and greater ease for different groups, such as the protocol described here.

Thus, there is a limitation in the field of neuropsychological rehabilitation when it comes to the topic of COVID-19 due to the lack of publications. In general, there are also few studies that evaluate the effectiveness of cognitive rehabilitation, even for other pathologies. It is important to develop more cognitive rehabilitation studies that target post-COVID patients with cognitive impairments, as well as other pathologies, in order to better understand the effectiveness of this therapeutic strategy for this specific population.

### Application experience

Participants in the experimental group showed motivation and engagement during the sessions, commenting on the positive role that rehabilitation had on them and how they perceived improvements in their daily lives. Some group participants developed friendships that extended into everyday life, which also facilitated the promotion of a bond and collaboration throughout the sessions. In addition, group interventions opened possibilities for identification among the participants, who reported feeling welcomed in the face of proximity to other people who had a similar demand. It could be observed that our data corroborate the scientific literature, according to the review^13^, which highlighted qualitative/subjective benefits such as improved well-being, self-confidence and perception of one’s own memory from the cognitive rehabilitation intervention.

### Ecological validity

The term “ecological validity” was defined by Brunswik, in 1955, as an experimental model whose results can be generalized to scenarios or events of everyday reality. For an instrument to be considered ecologically valid, it must present verisimilitude (how much it theoretically resembles the cognitive processes recruited by a routine activity) and/or veracity (how much an instrument is related to functional measures)^14^.

The instruments used in the application of the present protocol have little or no ecological validity; thus, the instructions and activities requested in each test are not very close to the patient’s reality, which is a limitation that has to do with the way this protocol was constructed. For the evaluation to be able to capture the changes and alterations promoted by the rehabilitation, it would be necessary for the activities proposed throughout each session to have a certain degree of similarity with the activities used in the assessment instrument; thus, it was not possible to elaborate a protocol that had greater ecological validity.

The development of appropriate scoring and interpretation methods, such as those already existing in standardized formal tasks, based on cognitive theories and psychometric criteria, is one of the main difficulties in the use of ecological tasks in neuropsychological assessment^13^. Thus, these characteristics must be considered when developing or studying ecological instruments. However, the successful development of these tools can provide more accurate cognitive diagnoses and more reliable means of assessing the outcomes of neuropsychological interventions.

In conclusion, despite the acknowledged limitation, the assessment and technique used in the present study are widely observed in studies published on the subject and can still be a parameter for improvement in relation to the cognitive performance and behavior of individuals.

Although many studies already emphasize the importance of this type of rehabilitation, there are few that bring a description of the protocol used; most only mention the techniques and strategies in a brief and superficial way, which makes it difficult for the professional and the students who are looking for content to rehabilitate their patients.

The present proposed protocol, which aims to train impaired cognitive abilities, mainly attention and memory, is in agreement with the few studies that are presented in the scientific literature. Moreover, cognitive rehabilitation has more consolidated evidence about its effectiveness for the treatment of other pathologies, so it can be estimated that it will also be a promising strategy for COVID-19.
